# Management of respiratory disorders in a Chinese medicine teaching clinic in Australia: review of clinical records

**DOI:** 10.1186/s13020-015-0063-8

**Published:** 2015-11-02

**Authors:** Wan Najbah Nik Nabil, Wenyu Zhou, Johannah Linda Shergis, Suzi Mansu, Charlie Changli Xue, Anthony Lin Zhang

**Affiliations:** Traditional and Complementary Medicine Research Program, Discipline of Chinese Medicine, School of Health Sciences, RMIT University, PO Box 71, Bundoora, VIC 3083 Australia

## Abstract

**Background:**

People seek Chinese medicine (CM) treatments for a variety of respiratory disorders, e.g., asthma and upper respiratory tract infection (URTI). No previous studies have reviewed the data available in medical records from Australian clinics. This study aims to identify the characteristics of patients with respiratory disorders who visited a CM teaching clinic at RMIT University in Melbourne, Australia.

**Methods:**

Primary data from January 2010 to December 2011 were collected from patient records in a CM teaching clinic at RMIT University. Patient data, including demographics, primary complaint, clinical history, lifestyle, CM treatment, and adverse events, were analyzed with descriptive statistics and the Chi square test using SPSS version 21.0.

**Results:**

From 1677 clinical records we identified 261 patients with respiratory disorders. The patients made a total of 842 visits (mean: 3.2 visits/patient; range: 1–34 visits) during the study period. The mean age of the patients was 38.5 ± 17.9 years, and the majority were female (65.5 %). The most common respiratory disorders were URTI (27.8 %), cough (20.8 %), hay fever or allergic rhinitis (18.6 %), sinus congestion (11.2 %), and asthma (7.6 %). Acupuncture was given at almost all visits (97.5 %) and was frequently combined with herbs (64.0 %). Fifteen adverse events were reported, but none were considered severe.

**Conclusion:**

In the CM teaching clinic, respiratory conditions were a common presenting complaint of patients, and were safely treated with a combination of acupuncture and herbs.

**Electronic supplementary material:**

The online version of this article (doi:10.1186/s13020-015-0063-8) contains supplementary material, which is available to authorized users.

## Background

While the Australian government spends more than AUD 3 billion annually on respiratory disorders, 19 % of Australian patients visited medical practitioners for respiratory disorders in 2007 and 2008 [1]. At least one-fifth of people with respiratory disorders, such as asthma and chronic obstructive pulmonary disease, seek complementary and alternative treatments, including acupuncture, herbal medicine, and lifestyle advice [2, 3]. However, little is known about the patient characteristics, clinical applications, and respiratory conditions treated in Chinese medicine (CM) clinics. The respiratory disorders included in this study were conditions affecting any part of the airways, including the nasal cavities, throat, larynx, pharynx, trachea, and lung [4].

Clinical records provide valuable data on the characteristics of patient populations in a health clinic [5] and provide insights into their therapies [6] and adverse events [7]. Knowledge of patient demographic characteristics would facilitate practitioners in including CM treatments [8]. Examination of documented adverse events can facilitate practitioners to learn about the risks of treatments [9], reduce adverse events [7], and report adverse events [9].

The RMIT University CM teaching clinic in Melbourne, Australia is open to the public. Patients are treated by senior students in their final undergraduate or postgraduate year, under the supervision of qualified CM practitioners registered with the CM Board of Australia. Available health services include needle acupuncture (with manual or electrical stimulation), laser acupuncture, Chinese herbal medicines (raw, granular, and manufactured), therapeutic massage, moxibustion, cupping, dietary advice, and lifestyle advice [10]. Chinese herb formulae are prepared in the clinic according to the prescription of the practitioners. At the time of the study, the patients were charged AUD 30 and 25 for initial and subsequent consultations, respectively. The consultation charges covered acupuncture treatment, therapeutic massage treatment, and cupping. However, the charges for Chinese herbal medicines varied and were dependent on the specific treatments [11].

In Australian CM clinics, the numbers of patients presenting with respiratory disorders are unknown. This study aims to identify the characteristics of patients with respiratory disorders who visited the RMIT CM teaching clinic.

## Methods

### Study settings

This study was a retrospective review and analysis of the clinical records of patients who received treatment for respiratory disorders at the RMIT University CM teaching clinic between January 2010 and December 2011. Patients signed informed consent forms on their first visit to the teaching clinic (Additional file 1). The signed consent form allowed students and staff at the CM clinic to use de-identified clinical records for learning and research purposes.

### Data source and data collection

Data were extracted consistently by WN using a predefined data extraction form in Microsoft Excel 2007 (Microsoft, Redmond, Washington, USA). The extraction process was piloted by WN and independently checked by WZ and AZ to ensure data quality. Full data were collected by WN and checked by WZ and AZ to ensure accuracy and reliability.

Identifiable patient data were not extracted from the clinical records to maintain privacy and confidentiality. A unique numerical coding system was applied when extracting the free-text data to ensure consistency. The patients consented to their records being used for medical research and both the data extraction form and the study protocol were approved by the RMIT University Human Research Ethics Committee (Project number: 02/12) (Additional file 2).

Clinical records were selected when a respiratory disorder was the patient’s primary complaint. Respiratory complaints included disorders of the airways including the nasal cavities, throat, larynx, pharynx, trachea, and lung [4]. The respiratory disorders were grouped according to the descriptions in the clinical records, including symptoms (e.g., runny nose) and diseases (e.g., hay fever, allergic rhinitis).

The extracted data included patient demographics (e.g., age, sex, occupation, and place of birth), primary complaint, medical history, current treatments and medications, alcohol intake, smoking history, physical activity, CM treatment, adverse events, and management of adverse events. A coding system was used to extract data on the CM treatment outcomes. Although we intended to assess patient responses to CM treatments, this was not feasible because the relevant data were not consistently recorded in the clinical records.

The original patient records did not record adverse events in a useable format for research purposes. Thus, WN extracted the events based on common terminology criteria outlined by the US National Institutes of Health [12] and evaluated the data using an intensity scale. Mild adverse events caused slight discomfort, moderate adverse events affected patients’ routine activities, and severe adverse events required therapeutic management.

### Data analyses

Data were expressed as mean ± standard deviation (SD) and frequency with percentage. The data were analyzed using SPSS software (IBM SPSS statistics for windows, version 21.0; IBM Corp., Armonk, NY, USA). Missing data were left blank and excluded from the analysis. Descriptive statistics were presented for patient characteristics including demographic data, primary complaint, and clinical history. The Chi square test, indicated with a degree of freedom (df), was used to compare the patterns of respiratory disorders. Values of *P* < 0.05 were considered to indicate statistically significant. Data on types of CM treatments, use of other treatment modalities, lifestyle advice, and adverse events were possible with multiple responses. For example, patients may have received a combination of CM treatments or advice or experienced more than one adverse event from a treatment.

## Results

### General demographics

The clinical records for a total of 1677 patients were assessed. Of these, 261 patients presented with primary respiratory disorders (Table 1). These 261 patients made a total of 842 visits (mean: 3.2 visits/patient; range: 1–34 visits). Females comprised 65.5 % of the patients. The mean age of the patients was 38.5 years (range: 2–100 years). Most of the patients were born in Australia (69.6 %), with the remaining patients were born in Asia (18.8 %), Europe (7.6 %), and other continents (4.0 %). The patients were students (38.7 %), employed (42.0 %), retired (13.6 %), housewives (3.7 %), and unemployed (2.1 %). Most patients lived within 10 km of the clinic (46.1 %), while 40.7 % lived 11–35 km away and 13.2 % lived further than 36 km. Almost one-third of the patients smoked (31.4 %) and approximately one-half were social alcohol drinkers (56.8 %). The majority of patients maintained regular exercise (78.0 %).

**Table 1:** Demographics of the patients

Item	n (%)
Number of patients	
Total	261 (100 %)
Gender	
Female	171 (65.5)
Male	90 (34.5)
Age (years)	
0–17	19 (7.3)
18–34	111 (42.5)
35–54	71 (27.2)
≥55	60 (23.0)
Country of birth	
Australia	174 (69.6)
Asia	47 (18.8)
Europe	19 (7.6)
Other	10 (4.0)
Missing	11
Occupation	
Student	94 (38.7)
Employed	102 (42.0)
Retired	33 (13.6)
Housewife	9 (3.7)
Unemployed	5 (2.1)
Missing	18
Distance from patients residence to the teaching clinic (km)	
≤10	119 (46.1)
11–35	105 (40.7)
≥36	37 (13.2)
Chinese medicine experience	
First time	63 (24.6)
Return user	193 (75.4)
Missing	5
Number of visits	
1	118 (45.2)
2–5	100 (38.3)
≥6	43 (16.5)
Number of other medical conditions	
None	140 (53.6)
1 or 2	43 (16.5)
More than 3	78 (29.9)
Use of other health care^a^	
Visit to medical practitioners	108 (89.3)
Supplements and herbs	27 (22.3)
Chiropractic	8 (6.6)
Homeopathy	5 (4.1)
Osteopathy	4 (3.3)
Massage	4 (3.3)
Naturopathy	4 (3.3)
Kinesiology	2 (1.7)
Myotherapy	1 (0.8)
Meditation	1 (0.8)
Physiotherapy	1 (0.8)
Qi gong	1 (0.8)
Podiatry	1 (0.8)
Buteyko exercise	1 (0.8)
Alcohol use (n = 44)	
Nondrinker or drink rarely	13 (29.5)
Drink socially	25 (56.8)
Drink daily	6 (13.6)
Smoking (n = 35)	
Non smoker	16 (45.7)
Ex-smoker	8 (22.9)
Smoker	11 (31.4)

Data analysis excludes missing values

^a^Patients’ use of other health care during January 2010 till December 2011. Patients may have used more than one other health care, total  % does not add up to 100 %

### Type and duration of respiratory disorders

The most common visits for respiratory disorders were for upper respiratory tract infection (URTI) (47.5 %), undiagnosed cough (15.7 %), hay fever or allergic rhinitis (14.6 %), sinus congestion (13.4 %), and asthma (3.1 %) (Table 2). There was a significant difference in the number of respiratory disorders between 2010 and 2011 (χ^2^ (df = 14) = 114.7, *P* < 0.001).

**Table 2:** Respiratory disorders

Respiratory disorder	Total patients, n (%) (N = 261)	Total visits, n (%) (N = 842)	Total visits 2010, n (%) (N = 363)	Total visits 2011, n (%) (N = 479)
URTI	124 (47.5)	234 (27.8)	121 (33.3)	113 (23.6)
Cough	41 (15.7)	175 (20.8)	74 (20.4)	101 (21.1)
Hay fever or allergic rhinitis	38 (14.6)	157 (18.6)	65 (17.9)	92 (19.2)
Sinus congestion	35 (13.4)	94 (11.2)	47 (12.9)	47 (9.8)
Asthma	8 (3.1)	64 (7.6)	8 (2.2)	56 (11.7)
Runny nose	3 (1.1)	13 (1.5)	3 (0.8)	10 (2.1)
Pulmonary fibrosis	2 (0.8)	22 (2.6)	0 (0)	22 (4.6)
Bronchitis	2 (0.8)	11 (1.3)	5 (1.4)	6 (1.3)
Shortness of breath	2 (0.8)	8 (1.0)	5 (1.4)	3 (0.6)
Pulmonary alveolar proteinosis	1 (0.4)	32 (3.8)	32 (8.8)	0 (0)
Sleep apnoea	1 (0.4)	12 (1.4)	0 (0)	12 (2.5)
Rhinitis	1 (0.4)	11 (1.3)	1 (0.3)	10 (2.1)
Chest infection	1 (0.4)	6 (0.7)	1 (0.3)	5 (1.0)
Pneumonia	1 (0.4)	2 (0.2)	0 (0)	2 (0.4)
Lung cancer	1 (0.4)	1 (0.1)	1 (0.3)	0 (0)
Total	261	842	363	479

Chi square test for significant difference between year 2010 and 2011: χ^2^ (14) = 114.7, *P* < 0.001

During the study period, 261 patients made a total of 842 respiratory disorder visits to the clinic

*URTI* upper respiratory tract infection

The duration of the respiratory condition was reported in 78.9 % of the clinical records. The reported durations were less than 2 weeks (36.7 %), 2–4 weeks (7.5 %), 5–51 weeks (13.5 %), 1–5 years (16.8 %), and >5 years (25.4 %).

Nearly one-half of the patients (*n* = 121; 46.3 %) used one or more other health services during the study period. Of these, medical practitioner visits were the most commonly used health service (89.3 % of patients). Other health services included supplements and herbs (22.3 %), chiropractic (6.6 %), homeopathy (4.1 %), osteopathy (3.3 %), massage (3.3 %), and naturopathy (3.3 %).

### CM treatments

Of the 261 patients with respiratory disorders, most visited the clinic once for their primary respiratory complaint (mean ± SD: 3.23 ± 3.95 visits; range: 1–34 visits). Comorbidities were also treated with CM as secondary complaints, and the most common secondary complaints were musculoskeletal and pain disorders (32.9 %), other respiratory disorders (17.3 %), migraine and headache (8.8 %), gastrointestinal disorders (6.5 %), and dermatological disorders (6.2 %). The majority of the patients (75.4 %) had previously experienced CM treatments.

In the 842 visits, the respiratory disorders were treated with acupuncture (97.5 %), Chinese herbs (74.2 %), cupping (7.5 %), massage (4.4 %), and moxibustion (1.1 %). The preparation types of the Chinese herbs were granules (40.7 %), manufactured (21.1 %), raw (14.1 %), and external-use wash or cream (0.7 %) (Table 3).

**Table 3:** Chinese medicine treatments

Chinese medicine treatment	Number of visits, n (%) (N = 842)
Type of treatment^a^	
Needle acupuncture	779 (92.5)
Granule herbs	343 (40.7)
Manufactured herbs	178 (21.1)
Raw herbs	119 (14.1)
Cupping	63 (7.5)
Ear acupressure	51 (6.1)
Laser acupuncture	38 (4.5)
Massage	37 (4.4)
Moxibustion	9 (1.1)
Externally applied herbs	6 (0.7)
Individual or combination treatment	
Individual treatment	190 (22.6)
Two types of treatment	528 (62.7)
Three of more types of treatment	123 (14.6)
No treatment	1 (0.1)
Advice (n = 116)^a^	
Diet	72 (62.1)
Exercise	34 (29.3)
Sleep, rest, relaxation or meditation	32 (27.6)
Increase water intake	25 (21.6)
Keep body warm	21 (18.1)
Other advice (such as personal hygiene, clothing, avoid allergen and stress environment)	18 (15.5)
Keep body covered from windy weather	9 (7.8)
Reduce coffee intake	8 (6.9)
Reduce alcohol intake	7 (6.0)
Reduce smoking	3 (2.6)
Total	842 (100 %)

^a^Patients may have had more than one type of treatment or advice at a single visit

At the majority of visits, patients were given both acupuncture and Chinese herbs (62.7 %). The most common combinations were needle acupuncture and granule herbs (46.2 %), needle acupuncture and manufactured herbs (22.9 %), needle acupuncture and raw herbs (17.8 %), laser acupuncture and granule herbs (4.0 %), and needle acupuncture and massage (1.7 %).

The combination of needle acupuncture and granule herbs was commonly used if the presenting conditions were pneumonia (100.0 %), lung cancer (100.0 %), pulmonary alveolar proteinosis (71.9 %), asthma (42.2 %), bronchitis (36.4 %), cough (30.9 %), sinus congestion (30.9 %), and URTI (27.4 %). Meanwhile, acupuncture treatment alone was more commonly used for pulmonary fibrosis (77.3 %), sleep apnea (50.0 %), runny nose (46.2 %), shortness of breath (37.5 %), and hay fever or allergic rhinitis (23.6 %).

A total of 214 different acupuncture points were used. The number of points used at each treatment ranged 2–15 (mean ± SD 6.3 ± 1.7; median: 6). The most frequently used acupuncture points were *he gu* (LI4; 43.8 %), *lie que* (LU7; 39.5 %), *zu san li* (ST36; 39.5 %), *feng long* (ST40; 35.1 %), and *ying xiang* (LI20; 31.3 %). The acupuncture point *ying xiang* (LI 20) was commonly used for sinus congestion (81.9 %), while *lie que* (LU7) was used for shortness of breath (62.5 %), *zu san li* (ST36) was used for bronchitis (72.7 %), and *ding chuan* (EX-B1) was used for asthma (56.3 %).

Overall, 68 different Chinese herbal formulae were used. In the Poisons Standard 2014, the Australian Therapeutic Goods Administration outlines that certain herbs like *Ephedra sinica* (*Ma Huang*) are grouped into Schedule 4, and can only be prescribed by authorized healthcare professionals and acquired from pharmacists upon prescription [13]. This study found that the clinic was fully compliant with the Poisons Standard 2014 by not prescribing, keeping, storing, or using herbs with toxicity. *Yu Ping Feng San* was the most commonly used formula (18.7 %), followed by *Xiao Qing Long Tang* (7.8 %) and *Zhi Sou San* (7.6 %). In total, 152 different herbs were used. The most commonly used herbs were *Radix Platycodi* (*Jie Geng*; 51.2 %), *Radix Glycyrrhizae* (*Gan Cao*; 41.0 %), *Rhizoma Atractylodis Macrocephalae* (*Bai Zhu*; 38.7 %), *Pericarpium Citri Reticulatae* (*Chen Pi*; 36.7 %), and *Radix Saposhnikoviae* (*Fang Feng*; 34.3 %). The formulae for the most common conditions were *Sang Ju Yin* for URTI (*n* = 18; 17.5 %), *Zhi Sou San* for cough (*n* = 26; 23.9 %), *Xin Yi San* for sinus congestion (*n* = 12; 25.0 %), and *Yu Ping Feng San* for hay fever or allergic rhinitis (*n* = 34; 47.9 %) and asthma (*n* = 28; 51.9 %). A total of 23 different manufactured products were prescribed, with the most common being *Bi Min Gan Wan* (28.1 %), *Yin Qiao San* (18.5 %), and *Qian Bai Bi Yan Pian* (11.2 %).

General health and wellbeing advice was documented at a relatively small number of visits (*n* = 116; 13.8 %). Lifestyle advice specific to CM comprised rest and meditation, protecting the body from windy weather, and general health approaches such as smoking cessation and exercise. Dietary advice included information on specific foods based on CM theory, and general dietary advice such as reducing coffee and alcohol, and increasing water intake (Table 3).

### Adverse events

There were 15 reported adverse events from the 842 visits (1.78 %). The 15 events were experienced by 11 patients (Table 4). 12 events were mild and caused minimal discomfort and three were moderate and caused some discomfort that interfered with daily activities, but none were severe. The adverse events included diarrhea or loose stool (three events), nausea or vomiting (two events), symptom aggravation (two events), skin rash (two events), hot sensation in the body (two events), headache (one event), dizziness (one event), stomach ache (one event), and bloating (one event). Potential causality was assessed to be related to the Chinese herbs except for one event of nausea and vomiting that was potentially related to acupuncture. The practitioners responded to the adverse events by modifying the patients’ treatment (e.g., changing the acupuncture points or changing the herbs or herb dosage; 53.3 %), stopping the treatment (33.3 %), or changing to another treatment (6.7 %).

**Table 4:** Adverse events

Adverse events	n (%)
Type of event	
Diarrhea or loose stools	3 (25.0)
Nausea and vomiting	2 (16.7)
Symptom aggravation	2 (16.7)
Skin rash	2 (16.7)
Hot sensation in the body	2 (16.7)
Headache	1 (8.3)
Dizziness	1 (8.3)
Stomach ache	1 (8.3)
Bloating	1 (8.3)
Practitioners’ action addressing adverse events	
Modify treatment regime	8 (53.3)
Stop the treatment	5 (33.3)
Provided a new Chinese medicine treatment	1 (6.7)
Missing data	1 (6.7)
Total	15 (100)

## Discussion

This study analyzed full clinical records from a CM teaching clinic in Australia with a specific focus on respiratory disorders. Young female students were identified as the main patient cohort visiting the teaching clinic for respiratory disorders. The most common respiratory conditions were URTI, cough, hay fever or allergic rhinitis, sinus congestion, and asthma, which are also the most common respiratory disorders encountered in Australian general practice [14]. Most visits were for acute episodes or symptoms that were present for less than 2 weeks. However, at least two-fifths of the patients had their condition for more than 1 year and attended the clinic for an acute flare-up. A large number of the visits involved treatment by both acupuncture and herbal medicine, suggesting that a combined CM intervention was common for the treatment of respiratory disorders.

Previous studies [15–18] reported that most patients were employed and middle-aged, while students and young people (age: 18–34 years) represented the largest proportion of patients in the present study. The teaching clinic examined is located on a university campus, and many of the patients were young students. The patients were more likely to be female, consistent with previous reports for studies on CM clinics [15–19].

The adverse event profile revealed a small number of mild and moderate adverse events, and was similar to the findings in an Australian survey on the safety of CM treatments [20]. None of the adverse events required specific intervention. However, in response to nearly half of the events, the CM treatment was modified.

This study had several limitations. The clinical records were developed for clinical purposes and their use for research and retrospective data collection was at times inconsistent and incomplete. Although the authors attempted to ensure accurate collection of information, misinterpretations or mistakes may have occurred. We could not assess the clear outcomes of the CM treatments. It was difficult to evaluate the treatment outcomes of patients owing to inconsistent documentation on their progress and the fact that some patients did not return to the clinic. In addition, the information relating to demographics, including age and employment, might change. Therefore, the results may not be generalizable or represent other clinical practices that are off-campus. The patients often used CM treatments in conjunction with other healthcare services. The clinical records did not reveal any interactions with other healthcare treatments, and the CM treatments appeared to be safe to use in this group of patients.

Future prospective studies on clinical records should be developed to meet the needs of both clinicians and researchers. These efforts could include the use of standardized clinical terminology, information on outcome-related data (e.g., validated quality of life or disease-specific questionnaires), and comprehensive documentation of adverse events.

The findings from this study may guide documentation of medical records (such as collecting data on treatment outcomes) [21], provide data for the planning of clinical research projects [22], and inform curriculum design for the CM learning program [22]. Moreover, understanding the profiles and behaviors of patients will promote the use of the clinic by potential patients and improve the treatment quality.

## Conclusion

In the CM teaching clinic, respiratory conditions were a common presenting complaint of patients, and were safely treated with a combination of acupuncture and herbs.

## Additional files

10.1186/s13020-015-0063-8 Patient consent form.
10.1186/s13020-015-0063-8 RMIT University Human Research Ethics Committee study approval.

## Abbreviations

CM: Chinese medicine
URTI: upper respiratory tract infections
