# Supplementary material for: Management of respiratory disorders in a Chinese medicine teaching clinic in Australia: review of clinical records
Source: Chin Med. 2015 Nov 2;10:31. doi: 10.1186/s13020-015-0063-8 (PMC4630930; doi:10.1186/s13020-015-0063-8)
Supplement: Supplementary file 1 — 10.1186/s13020-015-0063-8 Patient consent form. [file 13020_2015_63_MOESM1_ESM.pdf]

RMIT POLYCLINIC
BUNDOORA Chinese Medicine Teaching Clinic
A teaching clinic of RMIT University.

RMIT UNIVERSITY

Privacy Consent Form

The Division of Chinese Medicine and its teaching clinics are subject to new legislation aimed at protecting the privacy of personal information including health and medical records. As part of its function, the Division collects, uses and discloses such information. From time to time, it also uses this information, in a de-identified format, for research purposes. This document is a request to you to provide your consent to the use and disclosure of your personal information in certain circumstances.

I Mr./Mrs./Ms.________________________________understand that this clinic is a teaching clinic of RMIT University and in this role will collect certain personal information about me in relation to the diagnosis and/or treatment of a health-related condition. I hereby consent to the personal information in my file being used within the approved protocols of the Division of Chinese for case discussion, teaching, research, and examination purposes.

.... *Signature*

I also understand that I may revoke this permission at any time without prejudicing due care and treatment.

Signature ....

Date ....

(Signatures must be obtained in front of a witness - reception staff, clinician, student over 18)

Witness Signature ....

Date ....
