# Supplementary material for: Management of respiratory disorders in a Chinese medicine teaching clinic in Australia: review of clinical records
Source: Chin Med. 2015 Nov 2;10:31. doi: 10.1186/s13020-015-0063-8 (PMC4630930; doi:10.1186/s13020-015-0063-8)
Supplement: Supplementary file 2 — 10.1186/s13020-015-0063-8 RMIT University Human Research Ethics Committee study approval. [file 13020_2015_63_MOESM2_ESM.pdf]

RMIT UNIVERSITY

Human Research Ethics Committee (HREC)
Research and Innovation office

Notice of Approval

Date:	**13 March 2012**
Project number:	**02/12**
Project title:	***Public health issues related to Chinese medicine usage in respiratory diseases***
Risk classification:	**More than low risk**
Investigator:	**Wan Najbah Nik Nabil**
Approved:	From: **7 February 2012** To: **31 December 2012**

Terms of approval:

1. **Responsibilities of investigator**
   It is the responsibility of the above investigator to ensure that all other investigators and staff on a project are aware of the terms of approval and to ensure that the project is conducted as approved by HREC. Approval is only valid whilst investigator holds a position at RMIT University.
2. **Amendments**
   Approval must be sought from HREC to amend any aspect of a project including approved documents. To apply for an amendment use the request for amendment form, which is available on the HREC website and submitted to the HREC secretary. Amendments must not be implemented without first gaining approval from HREC.
3. **Adverse events**
   You should notify HREC immediately of any serious or unexpected adverse effects on participants or unforeseen events affecting the ethical acceptability of the project.
4. **Plain Language Statement (PLS)**
   The PLS and any other material used to recruit and inform participants of the project must include the RMIT university logo. The PLS must contain a complaints clause including the above project number.
5. **Annual reports**
   Continued approval of this project is dependent on the submission of an annual report.
6. **Final report**
   A final report must be provided at the conclusion of the project. HREC must be notified if the project is discontinued before the expected date of completion.
7. **Monitoring**
   Projects may be subject to an audit or any other form of monitoring by HREC at any time.
8. **Retention and storage of data**
   The investigator is responsible for the storage and retention of original data pertaining to a project for a minimum period of five years.

In any future correspondence please quote the project number and project title above.

A/Prof Barbara Polus
Chairperson
RMIT HREC

cc: Dr Peter Burke (Ethics Officer/HREC secretary), Dr Tony Zhang (supervisor).

K:\Governance\RMIT Ethics\HREC\HREC 2012\Correspondence\02-12 wan.doc
